# An Audit to Reevaluate the Adherence to the Guidelines in Patients With Urinary Tract Infection at the Al-Karak Hospital in Jordan

**DOI:** 10.7759/cureus.39509

**Published:** 2023-05-25

**Authors:** Sallam Alrosan, Mohammad Al Mse'adeen, Ibraheem M Alkhawaldeh, Ja’far Mishael, Nibal Aljarab'ah, Mohammad Aljarajreh, Mohannad Yamin, Mohammad Abu-Jeyyab

**Affiliations:** 1 Internal Medicine, Saint Luke’s Health System, Kansas City, USA; 2 School of Medicine, Mutah University, Al-Karak, JOR; 3 Internal Medicine, Al-Karak Hospital, Al-Karak, JOR

**Keywords:** urinary tract infection, hospital acquired uti, symptomatic uti, uti treatment, community acquired uti, simple uti

## Abstract

Background

Urinary tract infection (UTI) is a common and costly health problem that affects millions of people worldwide. The proper management of UTI requires adherence to clinical guidelines that are based on the best available evidence. However, compliance with these guidelines in real-world practice is often suboptimal.

Objective

This study is aimed to audit and reevaluate the adherence to the guidelines in UTI patients at Al-Karak Hospital, Jordan.

Methods

A retrospective cohort study was conducted. The first loop included 50 patients who presented with symptoms of simple uncomplicated UTI and were treated at the clinic during a three-month period. The second loop included a reevaluation of the first loop's findings after implementing changes to the clinical practice based on the initial audit results.

Results

The main factors that influenced the adherence were the type of UTI, the presence of comorbidities, the duration of hospitalization, and the antibiotic choice. At the first loop, the audit findings identified that the 100% standard National Institute for Health and Care Excellence (NICE) guidelines met the reach of 20 (40%) of the 50 patients. A revaluation of the audit findings identified that the 100% standard NICE guidelines met the reach of 36 of the 50 (72%) patients.

Conclusion

The study concluded that there is a need to improve adherence to the guidelines in UTI patients at the Al-Karak Hospital and suggested some recommendations to achieve this goal.

## Introduction

Urinary tract infections (UTIs) are a common and possibly hazardous condition that affects a large percentage of the population [1]. Accurate and timely diagnosis of UTIs is critical for effective treatment and prevention of complications [2]. Urine dipstick tests and visual examination of urine samples have traditionally been used to diagnose UTIs [3]. These tests, however, have limitations and can produce false negative results [4]. As a result, there is a growing interest in using urine culture as a diagnostic tool for UTIs [5]. According to the evidence gathered during this audit, using urine culture in the diagnosis of UTIs can provide more accurate results than traditional diagnostic methods such as urine dipstick tests and visual examinations [2]. This has the potential to benefit current patients by providing a more accurate diagnosis, leading to more effective treatment and better outcomes [4]. A urine culture can also benefit potential new patients by providing a more accurate and reliable diagnosis. Furthermore, the use of urine culture can benefit healthcare providers by lowering the risk of misdiagnosis and ensuring that patients receive the best treatment possible [5].

## Materials and methods

This clinical audit aimed to assess adherence to the National Institute for Health and Care Excellence (NICE) guidelines regarding urine analyses and culture in UTI management at the Al-Karak Governmental Hospital (KGH) between January 2023 and March 2023. The audit was conducted in two loops. The ﬁrst loop included those who presented with symptoms of UTI and were treated at the clinic during a three-month period (see appendix). The second loop included a reevaluation of the ﬁrst loop's ﬁndings after implementing changes to the clinical practice based on the initial audit results.

The NICE has published guidelines on the diagnosis of UTIs. These guidelines recommend that pregnant women and men should have a midstream urine sample sent for culture and susceptibility testing. For those under the age of 16 years, a urine sample can be sent for culture and susceptibility testing, or a dipstick test can be used.

UTI is diagnosed in women under the age of 65 years who have two or more key urinary symptoms and no other excluding causes or warning signs. Key urinary symptoms include painful urination, frequent urination, urge to urinate, burning sensation during urination, and cloudy or bloody urine. Excluding causes or warning signs include sexually transmitted infections, kidney stones, and bladder cancer.

Dipstick testing is not available for adults who have indwelling urinary catheters. Antibiotics are not given to men or non-pregnant women to treat asymptomatic bacteriuria, which is the presence of bacteria in the urine without any symptoms. Non-pregnant women with an uncomplicated lower UTI are given a three-day course of antibiotics, while men and pregnant women are given a seven-day course of antibiotics. Men with recurrent UTIs and women with recurrent lower UTIs where the cause is unknown or recurrent upper UTIs are referred to a specialist. These guidelines provide healthcare professionals with clear guidance on the diagnosis and treatment of UTIs. By following these guidelines, healthcare professionals can help to ensure that patients receive the correct diagnosis and treatment, which can help to improve their quality of life.

We conducted a retrospective cohort study using data from patients' medical records. The audit criteria were based on NICE guidelines, which recommend carrying out urine culture and susceptibility testing for patients with symptoms of UTI, prior to antibiotic treatment. The guidelines also advise against using urine dipstick tests as a substitute for urine culture and susceptibility testing in these patients. Furthermore, the guidelines suggest repeating urine culture and susceptibility testing in patients with persistent symptoms or recurrent UTIs. The audit also assessed the types of antibiotics prescribed for UTI treatment. The data were analyzed using the Jamovi® software, which provides a user-friendly interface for statistical analysis.

Based on the first loop's findings, changes were made to clinical practice to improve adherence to NICE guidelines. These changes included regular staff training on NICE guidelines, the implementation of a standardized process for collecting urine samples, and improved communication between clinicians and laboratory staff.

In the second loop, a reevaluation was conducted using the same audit criteria and data collection methods as in the first loop. The findings of the second loop were analyzed, and the results were compared to those of the first loop to determine if the changes made to clinical practice had a positive impact on adherence to NICE guidelines.

Ethical considerations

Before conducting the audit, ethical considerations were taken into account. The study was approved by the hospital's ethical committee, and the patients' confidentiality was maintained throughout the data collection process. All patient data were de-identified, and only authorized personnel had access to the data. Patients' rights to privacy and autonomy were respected, and their medical records were accessed only for audit purposes.

Limitations of this audit include the small sample size and the retrospective data collection method. Future audits could involve a larger sample size and prospective data collection to provide more robust findings.

## Results

First loop

**Figure 1:**
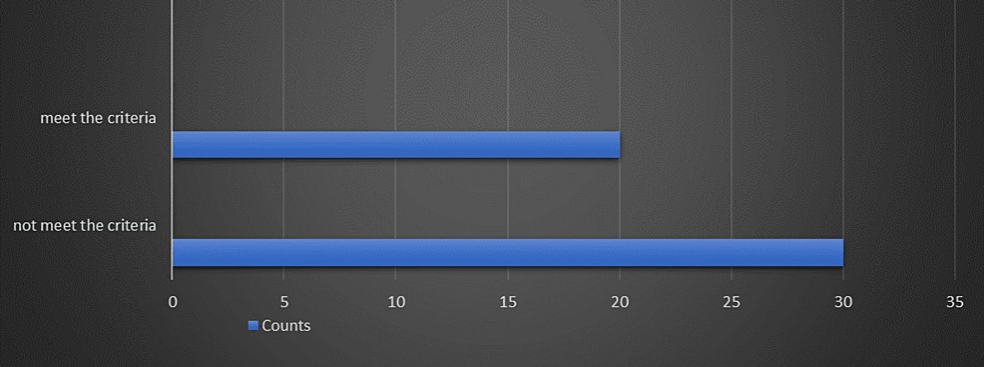
The frequency of the number of samples that are taken from patients

At the first loop, the audit findings identified that the 100% standard NICE guidelines met the reach of 20 (40%) of the 50 patients (Table 1 and Figure 1). Of them, 38 were females and 12 were males.

**Table 1:** Frequencies of the number of samples that are taken from patients

Number of samples that are taken from patients	Counts	% of Total	Cumulative %
Not met the criteria	30	60.0%	60.0%
Met the criteria	20	40.0%	100.0%

**Figure 2:**
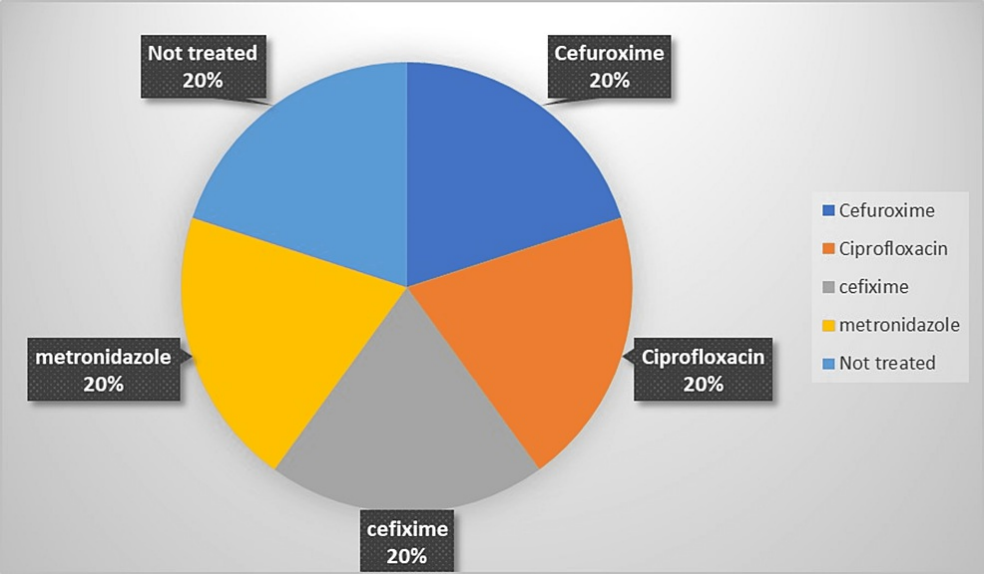
The frequency of drugs prescribed for the included patient This figure shows the percentages of 50 patients included in the study.

Also, 10 (20%) of them were treated with ciprofloxacin, 10 (20%) of them were treated with cefixime, 10 (20%) were treated with metronidazole, 10 (20%) of them were treated with cefuroxime, and 10 (20%) of them are not treated (Table 2 and Figure 2).

**Table 2:** Frequencies of drugs

Drug	Counts	% of Total	Cumulative %
Cefuroxime	10	20.0%	20.0%
Ciprofloxacin	10	20.0%	40.0%
Cefixime	10	20.0%	60.0%
Metronidazole	10	20.0%	80.0%
Not treated	10	20.0%	100.0%

Second loop

**Figure 3:**
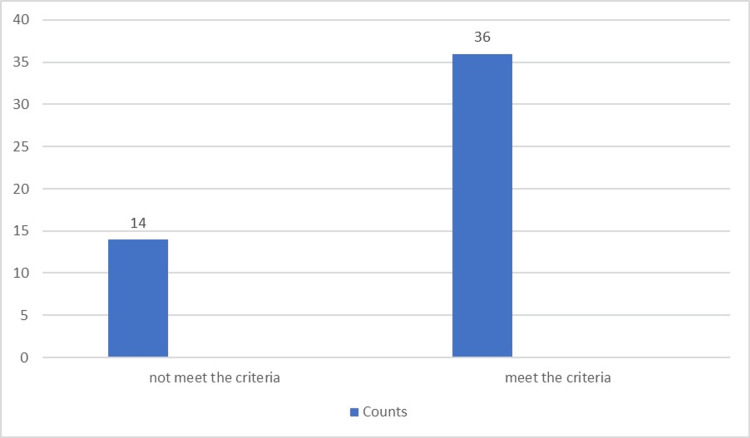
The frequency of the number of samples that are taken from patients at the second loop

A reevaluation of the audit findings identified that the 100% standard NICE guidelines met the reach of 36 of the 50 (72%) patients (Table 3 and Figure 3).

**Table 3:** Frequencies of the number of samples that are taken from patients

Number of samples that are taken from patients	Counts	% of Total	Cumulative %
Not met the criteria	14	28.0%	28.0%
Met the criteria	36	72.0%	100.0%

## Discussion

UTI is a common microbial infection that causes inflammation of the urinary tract in people of all ages and genders. These infections can range from minor bladder inflammation (cystitis) to life-threatening uroseptic shock. UTI is the most common infection that results in an antibiotic prescription following a doctor's visit [6].

An uncomplicated UTI is a bacterial infection of the bladder and associated structures, which occurs in patients who do not have any structural abnormalities or comorbidities such as diabetes, immunocompromised state, or pregnancy. Without symptoms, bacteriuria does not constitute a UTI [7].

Pain during urination (dysuria), frequent urination (frequency), inability to start the urine stream (hesitancy), sudden onset of the need to urinate (urgency), and blood in the urine are symptoms of uncomplicated UTIs (hematuria). Patients with uncomplicated UTIs typically do not have a fever, chills, nausea, vomiting, or back pain, all of which are symptoms of kidney involvement or upper tract disease/pyelonephritis [8].

The vast majority of UTIs are caused by *E. coli*, followed by *Klebsiella*, but other organisms of importance include *Proteus*, *Enterobacter*, and *Enterococcus *[9].

Diagnostic failure in bacterial infections is the leading cause of improper antibiotic use, treatment delays, and low survival rates in septic conditions. As a result, the most important requirements for preventing complicated UTI conditions such as urosepsis are early diagnosis and appropriate antibiotic therapy [6].

Epidemiology

UTIs are among the most common bacterial infections in women. They typically occur between the ages of 16 and 35 years, with 10% of women infected annually and more than 40%-60% infected at least once in their lives. Recurrences are common, with nearly half of those infected getting another infection within a year. Females are at least four times more likely than males to get UTIs [10,11].

Risk factors

UTIs are more common in females because their urethras are shorter and closer to the rectum. Previous UTIs, sexual activity, and obesity are all risk factors for UTIs. Changes in the bacteria that live inside the vagina or vaginal flora (for example, menopause or the use of spermicides can cause these bacterial changes), pregnancy, age (older adults and young children are more likely to get UTIs), and poor hygiene are all risk factors for UTIs [12]. The use of a urinary catheter is a major risk factor for UTIs. Frequent pelvic exams and the presence of urinary tract anatomical abnormalities can also predispose one to a UTI [9]. People who urinate and empty their bladder frequently are less likely to develop a UTI [13].

Complications

Complications of UTIs include persistent lower urinary tract symptoms, staghorn urinary calculi, pyelonephritis, emphysematous pyelonephritis and cystitis, incontinence, focal renal nephronia, renal abscess, chronic prostatitis, prostatic abscess, hypertension, and renal failure [7].

Outcomes

The majority of women who have a UTI have a positive outcome. The duration of symptoms after antibiotic treatment is two to four days. Unfortunately, nearly 30% of women will have the infection recur. Morbidity is more common in elderly patients or those with renal calculi. Other risk factors for recurrence include diabetes, underlying malignancy, chemotherapy, and chronic bladder catheterization. The mortality rate following a UTI is close to zero [14-16].

Discussion of results

From September 1, 2022, to the end of September 2022, 50 outpatient and inpatient cases were collected from all hospital departments including the emergency department and outpatient clinics, of which 38 patients were females and 12 were males. We found that 40% of the patients (20 patients) met the 100% standard NICE guidelines for the diagnosis of UTI. To assess if any changes were implanted, a re-audit was done, which showed that 72% of the collected data (36 patients) met the current standards that have been set (improvement in terms of diagnostic accuracy of UTIs). However, the ideal of 100% was still not achieved.

This may be attributed to the absence of established uniﬁed guidelines for UTI diagnosis in KGH, low orientation of the doctors with UTI diagnosis according to the NICE guidelines, missing data in the computer system, especially for patients who did their tests in private labs, uncooperative patients in terms of doing urine culture tests, or improper collection of urine samples.

Suggestions for improvement include setting clear uniﬁed guidelines to be followed in KGH for UTI diagnosis, informing all departments about them, and incorporating those guidelines into computer systems to make it easier for doctors to follow. All private lab results should be included in the computer system by doctors or the tests should be repeated in the hospital lab.

Increasing clinicians' awareness of the importance of using urine culture as a diagnostic tool for UTIs can be done through regular presentations and audits about the same topic to improve and assess the response of doctors toward this important topic.

Regarding treatment, 80% of patients (40 patients) were treated with antibiotics such as ciprofloxacin, cefixime, metronidazole, and cefuroxime for every 10 patients, respectively, whereas 20% of patients (10) were not treated. This may raise the problem of inaccurate diagnosis of UTI as a result of not using urine culture as a diagnostic tool due to the absence of clear information available to doctors about UTI diagnosis policy available in the hospital and high load tasks on the doctors during the duty time. Suggestions for improvement include setting urine culture tests as a default option when ordering urinalysis tests in the computer system.

## Conclusions

This study backs up the theory that using urine culture to diagnose UTIs can provide more accurate results than traditional diagnostic methods such as urine dipstick tests and visual examinations. It is recommended that healthcare workers emphasize the significance of urine culture as a diagnostic tool for UTIs as this should improve the diagnostic accuracy and allow for more targeted antibiotic therapy.

## Appendices

**Table 4:** First loop patients' demographic characteristics and presenting symptoms UTI: Urinary tract infection; DM: Diabetes mellitus; OB/GYN: Obstetrician–gynecologist; M: Male; F: Female.

S. No	Gender	Age (Years)	Chief Complaints
1	M	63	Follow-up after bladder injury
2	M	79	After the cardiac cath.
3	F	64	Supra pubic pain (SPP) + hesitancy
4	F	62	SPP
5	F	62	SPP flank pain
6	M	23	Hematuria
7	M	10	SPP
8	M	40	Right flank pain
9	F	39	SPP
10	F	15	SPP + dysuria
11	F	68	SPP
12	M	62	SPP + hesitancy
13	F	50	SPP dysuria
14	M	52	Flank pain
15	M	38	Hesitancy dysuria + SPP
16	F	15	SPP dysuria
17	M	27	Frequency, hesitancy, and dysuria
18	F	38	SPP dysuria
19	F	37	Frequency
20	F	59	Fever + SPP
21	M	40	SPP
22	F	83	Femoral fracture
23	F	36	OB/GYN exam
24	F	43	Placenta previa
25	F	26	OB/GYN exam
26	F	22	Preterm labor
27	F	28	OB/GYN exam
28	F	33	Delivery
29	F	22	Abdominal pain
30	F	26	Back pain dysuria
31	F	31	Gestational DM
32	F	85	Back pain
33	M	41	UTI
34	M	55	Loin pain
35	F	6	Abdominal pain
36	F	68	Hysterectomy
37	F	24	High-risk pregnancy
38	F	49	Lower abdominal pain
39	F	42	DM
40	F	30	Lower abdominal pain
41	F	38	Headache
42	F	31	Follow-up after surgery
43	F	38	Lower back pain
44	F	43	Follow-up after surgery
45	F	30	Post-cesarian section
46	F	41	UTI
47	F	26	Dilated cervix
48	F	26	Missed abortion
49	F	46	Post hysterectomy
50	F	51	UTI
